# Treatment of Middle East Respiratory Syndrome with a combination of lopinavir-ritonavir and interferon-β1b (MIRACLE trial): study protocol for a randomized controlled trial

**DOI:** 10.1186/s13063-017-2427-0

**Published:** 2018-01-30

**Authors:** Yaseen M. Arabi, Adel Alothman, Hanan H. Balkhy, Abdulaziz Al-Dawood, Sameera AlJohani, Shmeylan Al Harbi, Suleiman Kojan, Majed Al Jeraisy, Ahmad M. Deeb, Abdullah M. Assiri, Fahad Al-Hameed, Asim AlSaedi, Yasser Mandourah, Ghaleb A. Almekhlafi, Nisreen Murad Sherbeeni, Fatehi Elnour Elzein, Javed Memon, Yusri Taha, Abdullah Almotairi, Khalid A. Maghrabi, Ismael Qushmaq, Ali Al Bshabshe, Ayman Kharaba, Sarah Shalhoub, Jesna Jose, Robert A. Fowler, Frederick G. Hayden, Mohamed A. Hussein, And the MIRACLE trial group, Yaseen M. Arabi, Adel Alothman, Hanan H Balkhy, Abdulaziz Al-Dawood, Sameera AlJohani, Shmeylan Al Harbi, Suleiman Kojan, Majed Al Jeraisy, Ahmad M. Deeb, Jesna Jose, Mohamed A. Hussein, Mohammed Al Muhaidib, Musharaf Sadat, Hala Al Anizi, Reggie Dael, Abdullah M Assiri, Mohammad AlMazroa, Ayed Asiri, Ziad A Memish, Sameeh S Ghazal, Sarah H Alfaraj, Fahad Bafaqeeh, Abdulrahman Al Harthy, Mohammed Al Sulaiman, Ahmed Mady, Yasser Mandourah, Ghaleb. A. AlMekhlafi, Nisreen Murad Sherbeeni, Fatehi Elnour Elzein, Reema Muhammed, Shatha Al Samirrai, Shatha Awad, Rylen Cabio Cabal, Abdulrauf Ahmad Malibary, Bander Al Onazi, Maha Aljuhani, Melven Vince, Abdullah Almotairi, Mushira Al Enani, Alaa Alqurashi, Fatimah Alenezi, Nada Alkhani, Khalid A. Maghrabi, Fahad Al-Hameed, Asim AlSaedi, Abdulhakeem Thaqafi, Ohoud Al Oraabi, Jalal Rifai, Pansy Elsamadisi, Medhat S. Hendy, Sara AbuBaker Basher, Muhammed Abduldhaher, Wael Bajhamoum, Ismael Qushmaq, Sarah Shalhoub, Yusri Taha, Javed Memon, Shahinaz Bashir, Ibraheem Al-Dossary, Saleh Al Mekhloof, Bader Al-Muhainy, Shehab Suliman, Mohammed S. Alshahrani, Ali Al Bshabshe, Ayman Kharaba, Ahmad Al Jabri, Magdy Farid, Alawi Alaidarous, Wael Alseraihi, Husam Shahada, Jinish Shimi, Syed Riaz, Bader Alharthi, Osama Yasin, Mohammad Khathlan, Robert A. Fowler, Frederick G. Hayden

**Affiliations:** 1 College of Medicine, King Saud Bin Abdulaziz University for Health Sciences, King Abdullah International Medical Research Center, Riyadh, Saudi Arabia; 2 Intensive Care Department, King Abdulaziz Medical City, Riyadh, Saudi Arabia; 3 Department of Medicine, King Abdulaziz Medical City, Riyadh, Saudi Arabia; 4 Department of Infection Prevention and Control, King Abdulaziz Medical City, Riyadh, Saudi Arabia; 5 Department of Pathology and Laboratory Medicine, King Abdulaziz Medical City, Riyadh, Saudi Arabia; 6 College of Pharmacy, King Saud Bin Abdulaziz University for Health Sciences, King Abdullah International Medical Research Center, Riyadh, Saudi Arabia; 7 Pharmaceutical Care Department, King Abdulaziz Medical City, Riyadh, Saudi Arabia; 8 Infection Prevention and Control, Ministry of Health, Riyadh, Saudi Arabia; 9 College of Medicine, King Saud Bin Abdulaziz University for Health Sciences, King Abdullah International Medical Research Center, Jeddah, Saudi Arabia; 10 Intensive Care Department, King Abdulaziz Medical City, Jeddah, Saudi Arabia; 11 Department of Infection Prevention and Control, King Abdulaziz Medical City, Jeddah, Saudi Arabia; 12 Department of Intensive Care Services, Prince Sultan Military Medical City, Riyadh, Saudi Arabia; 13 Infectious Diseases Division, Prince Sultan Military Medical City, Riyadh, Saudi Arabia; 14 Intensive Care Section, Department of Medicine, King Abdulaziz Hospital, Alahsa, Saudi Arabia; 15 Department of Medicine, King Abdulaziz Hospital, Alahsa, Saudi Arabia; 16 Department of Critical Care Medicine, King Fahad Medical City, Riyadh, Saudi Arabia; 17 Department of Critical Care Medicine, King Faisal Specialist Hospital and Research, Riyadh, Saudi Arabia; 18 Department of Medicine, King Faisal Specialist Hospital and Research Center, Jeddah, Saudi Arabia; 19 Department of Critical Care Medicine, King Khalid University, Aseer Central Hospital, Abha, Saudi Arabia; 20 Department of Critical Care, King Fahad Hospital, Ohoud Hospital, Al-Madinah Al-Monawarah, Saudi Arabia; 21 Department of Medicine, King Fahad Armed Forces Hospital, Jeddah, Saudi Arabia; 22 Department of Biostatistics and Bioinformatics, King Saud Bin Abdulaziz University for Health Sciences, King Abdullah International Medical Research Center, Riyadh, Saudi Arabia; 23 AMR Infection Control and Publications AIP/PED/HSE/HQ, Institute of Health Policy Management and Evaluation, University of Toronto, Toronto, ON Canada; 24 Department of Critical Care Medicine, Sunnybrook Hospital, 2075 Bayview Avenue, Room D478, Toronto, ON Canada; 25 Department of Medicine, Sunnybrook Hospital, 2075 Bayview Avenue, Room D478, Toronto, ON Canada; 26 Department of Medicine, Division of Infectious Diseases and International Health, University of Virginia School of Medicine, Charlottesville, VA USA; 27 International Severe Acute Respiratory and Emerging Infection Consortium (ISARIC) University of Oxford, Old Road Campus, Roosevelt Drive, Oxford, OX3 7FZ UK

**Keywords:** Coronavirus, MERS, Antiviral, Saudi Arabia, Clinical trial, Lopinavir/ritonavir, Interferon-β1b

## Abstract

**Background:**

It had been more than 5 years since the first case of Middle East Respiratory Syndrome coronavirus infection (MERS-CoV) was recorded, but no specific treatment has been investigated in randomized clinical trials. Results from in vitro and animal studies suggest that a combination of lopinavir/ritonavir and interferon-β1b (IFN-β1b) may be effective against MERS-CoV. The aim of this study is to investigate the efficacy of treatment with a combination of lopinavir/ritonavir and recombinant IFN-β1b provided with standard supportive care, compared to treatment with placebo provided with standard supportive care in patients with laboratory-confirmed MERS requiring hospital admission.

**Methods:**

The protocol is prepared in accordance with the SPIRIT (Standard Protocol Items: Recommendations for Interventional Trials) guidelines. Hospitalized adult patients with laboratory-confirmed MERS will be enrolled in this recursive, two-stage, group sequential, multicenter, placebo-controlled, double-blind randomized controlled trial. The trial is initially designed to include 2 two-stage components. The first two-stage component is designed to adjust sample size and determine futility stopping, but not efficacy stopping. The second two-stage component is designed to determine efficacy stopping and possibly readjustment of sample size. The primary outcome is 90-day mortality.

**Discussion:**

This will be the first randomized controlled trial of a potential treatment for MERS. The study is sponsored by King Abdullah International Medical Research Center, Riyadh, Saudi Arabia. Enrollment for this study began in November 2016, and has enrolled thirteen patients as of Jan 24-2018.

**Trial registration:**

ClinicalTrials.gov, ID: NCT02845843. Registered on 27 July 2016.

**Electronic supplementary material:**

The online version of this article (doi:10.1186/s13063-017-2427-0) contains supplementary material, which is available to authorized users.

## Background

Since the first report of infection with the Middle East Respiratory Syndrome coronavirus (MERS-CoV) was published in 2012, the World Health Organization has been notified of 2123 laboratory-confirmed infections with MERS-CoV from 27 countries and at least 740 reported deaths (case fatality 35%) [1]. There is no specific therapy of proven efficacy available and no potential treatment has been tested in a randomized clinical trial (RCT) for evaluation [2–4]. Based on results from in vitro and animal studies of MERS-CoV infection, the guanosine analog ribavirin, in combination with interferon alpha (IFN-α), has been used to treat patients with MERS [5–8]. However, the concentration of ribavirin required to inhibit MERS-CoV in vitro exceeds peak levels in the blood after therapeutic doses in humans [9–11]. Furthermore, retrospective studies with IFN-α2a, IFN-α2b or IFN-β1a in combination with ribavirin have not shown a clear benefit in patients with MERS [6–8, 12].

MERS-CoV attenuates the interferon (IFN) response of the innate immune system, and this mechanism is thought to impair the antiviral adaptive type 1 T-helper cell (Th-1) immune response [13–15]. In vitro, IFN-α and IFN-β have inhibitory effects on MERS-CoV and on the closely related coronavirus, Severe Acute Respiratory Syndrome (SARS)-CoV, but MERS-CoV appears to be significantly more sensitive than SARS-CoV to IFN effects [15, 16]. Among IFN subtypes, IFN-β1b causes the greatest in vitro inhibition of MERS-CoV, and has a significantly lower ratio of the half-maximal effective concentration (EC) to the maximal plasma concentration (EC_50_:C_max_) than IFN-α2a, IFN-α2b or IFN-β1a, indicating potentially greater efficacy [9, 11]. This observation may also partially explain the lack of clear benefit associated with the use of IFN-α2a, IFN-α2b or IFN-β1a in combination with ribavirin. Recombinant IFN-β1b (Betaseron®, Bayer, Leverkusen, Germany) is approved for multiple sclerosis [17–19].

Based on in vitro data, the combination of lopinavir and ritonavir has been considered as a candidate therapy for MERS. Lopinavir and ritonavir are antiretroviral protease inhibitors used in combination for the treatment of human immunodeficiency virus (HIV) infection and have limited side effects [20]. The combination of lopinavir/ritonavir (Kaletra®, Abbott Laboratories, Chicago, IL, USA) has also been used for the treatment of SARS. In one study, the combination of lopinavir/ritonavir used in 41 patients with SARS was associated with significantly fewer adverse clinical outcomes (acute respiratory distress syndrome or death) 21 days after the onset of symptoms compare to ribavirin alone used in 111 historical controls (2.4% versus 28.8%, *p* = 0.001) [21]. However, the historical nature of the control comparison does not allow for a valid estimate of efficacy. In a high-throughput screening for antiviral compounds, lopinavir inhibited the replication of MERS-CoV at levels below those that occur in the circulation after a single oral dose of lopinavir/ritonavir (400 mg lopinavir with 100 mg ritonavir), suggesting that the drug can achieve therapeutic levels in vivo [16, 22]. The effects of lopinavir/ritonavir, IFN-β1b and mycophenolate mofetil (MMF), all of which have shown viral inhibitory effects in vitro, have been tested in common marmosets with severe MERS-CoV infection [23]. The animals treated with lopinavir/ritonavir or IFN-β1b had improved clinical, radiological, pathological and viral-load outcomes compared with untreated animals. By contrast, treatment with MMF resulted in severe or fatal disease, with higher mean viral loads than in untreated animals. Untreated animals and MMF-treated animals had a mortality of 67% by 36 h compared to 0–33% among animals treated with lopinavir/ritonavir or IFN-β1b [23].

In one case from the 2015 Korean MERS outbreak, lopinavir/ritonavir used in combination with ribavirin and IFN-α2a resulted in defervescence, virological clearance and survival [24]. In another case from Greece, lopinavir/ritonavir combined with IFN and ribavirin was started on day 13 of a MERS illness and was followed 2 days later by clearance of viremia, although viral ribonucleic acid (RNA) persisted in the respiratory secretions until the fourth week of illness and the patient subsequently died [25]. During the Korean outbreak of MERS, most patients that developed respiratory illness received triple antiviral therapy composed of pegylated interferon (IFN)-α, ribavirin, and lopinavir/ritonavir; however, data about the efficacy of this approach are lacking [26]. These findings, together with the availability and safety profiles of lopinavir/ritonavir and IFN-β1b, suggest that the combination of these agents has potential efficacy for the treatment of patients with MERS.

The objective of the MIRACLE trial (the *M*ERS-CoV *I*nfection t*R*eated with *A C*ombination of *L*opinavir/ritonavir and int*E*rferon-β1b) is to assess the efficacy of administering a combination of lopinavir/ritonavir and recombinant IFN-β1b to hospitalized adults with laboratory-confirmed MERS. The study is designed as recursive, two-stage, group sequential, multicenter, randomized, placebo-controlled, double-blind trial. We report the study protocol according to the SPIRIT (Standard Protocol Items: Recommendations for Interventional Trials) template [27].

## Methods

### Study design

The study is a recursive, two-stage, group sequential, multicenter, randomized, placebo-controlled, double-blind trial [28]. The trial is initially designed with 2 two-stage components with two interim analyses and one final analysis. The first two-stage component is designed to adjust sample size and determine futility stopping, but not efficacy stopping. The second two-stage component is designed to determine efficacy stopping and possibly readjustment of sample size to maintain conditional power at the final analysis.

### Study population

#### Inclusion criteria at eligibility assessment

1. Adult (defined as ≥ 18 years of age)
2. Laboratory confirmation of MERS-CoV infection by reverse-transcription polymerase chain reaction (RT-PCR) from any diagnostic sampling source, and
3. New organ dysfunction that is judged to be related to MERS including: hypoxia defined as requirement of supplemental oxygen to maintain oxygen saturations > 90%, hypotension (systolic blood pressure < 90 mmHg) or the need for vasopressor/inotropic medication, renal impairment (increase of creatinine by 50% from baseline, glomerular filtration rate reduction by > 25% from baseline or urine output of < 0.5 ml/kg for 6 h – risk stage by RIFLE criteria) [29], neurological pathology (reduction of Glasgow Coma Scale by 2 or more, i.e., 13 or lower of 15 points), thrombocytopenia (<150,000 platelets/mm^3^) or gastrointestinal symptoms that require hospitalization (e.g., severe nausea, vomiting, diarrhea or/and abdominal pain).

#### Exclusion criteria at eligibility assessment

1. Suicidal ideation based on history (contraindication to IFN-β1b)
2. Known allergy or hypersensitivity reaction to lopinavir/ritonavir or to recombinant IFN-β1b, including, but not limited to, toxic epidermal necrolysis, Stevens-Johnson syndrome, erythema multiforme, urticaria or angioedema
3. Elevated alanine aminotransferase (ALT) more than five-fold the upper limit in the hospital’s laboratory
4. Use of medications that are contraindicated with lopinavir/ritonavir and that cannot be replaced or stopped during the study period, such as CYP3A inhibitors (see Table 1)

**Table 1:** Lopinavir/ritonavir interactions with drugs commonly used in the intensive-care unit

Drug	Possible interaction	Management	Action at enrollment	Action after enrollment
Amiodarone	Increased risk of amiodarone toxicity (hypotension, bradycardia, sinus arrest). Increased QT-interval prolongation	Concurrent use is contraindicated	At the time of enrollment, amiodarone therapy with no alternative is an exclusion criterion	Consider alternatives to amiodarone. If no alternative to amiodarone is available, consider using a reduced dose. Monitor for increased amiodarone serum concentrations, altered liver function test results and evidence of QT-interval prolongation
Fentanyl	Concurrent use of fentanyl and CYP3A4 inhibitors may result in an increased risk of fentanyl toxicity, resulting in fatal respiratory depression	In non-mechanically ventilated patients, concurrent use is contraindicated. In mechanically ventilated patients, avoid fentanyl or use reduced doses	Consider alternatives to fentanyl. Use lower doses and adjust the dose to target analgesia and sedative effects	Consider alternatives to fentanyl. Use lower doses and adjust the dose to target analgesia and sedative effects
Fluconazole	Increased ritonavir exposure and risk of QT-interval prolongation	Avoid concomitant use if possible If fluconazole is required, closely monitor electrocardiogram for QT-interval prolongation	Use alternatives to fluconazole	Use alternatives to fluconazole. Fluconazole-mediated CYP3A4 inhibition may continue for 4–5 days after discontinuation because of its long half-life
Midazolam	Increased midazolam plasma concentrations, which can lead to midazolam toxicity	In non-mechanically ventilated patients, concurrent use is contraindicated. In mechanically ventilated patients, avoid use of midazolam if possible. If needed, use reduced midazolam doses and monitor effects	Consider alternatives to midazolam. Use lower doses and adjust the dose to target sedative effects	Consider alternatives to midazolam. Use lower doses and adjust the dose to target sedative effects. If the concomitant use of midazolam and lopinavir/ritonavir is required, closely monitor patients for midazolam adverse effects (excessive sedation, confusion and respiratory depression) and consider using a reduced midazolam dose
Quetiapine	Increased risk of QT-interval prolongation, *torsades de pointes* or other notable ventricular tachyarrhythmias	Concomitant administration is contraindicated	Use alternatives to quetiapine	Use alternatives to quetiapine. If concomitant use is required, reduce the quetiapine dose to one sixth of the standard dose, and when the lopinavir/ritonavir is discontinued, the dose of quetiapine should subsequently be increased to the standard dose
Rifampin	Decreased lopinavir/ritonavir plasma concentrations. Rifampin may enhance the toxic effect of lopinavir, specifically increasing the risk of hepatocellular toxicity	Concurrent use is contraindicated	If concomitant use is required, rifabutin 150 mg every other day or 150 mg three times a week is recommended for concomitant use with lopinavir/ritonavir	If concomitant use is required, rifabutin 150 mg every other day or 150 mg three times a week is recommended for concomitant use with lopinavir/ritonavir
Sildenafil	Increased sildenafil plasma levels, thereby increasing the risk for sildenafil adverse effects (hypotension, visual changes and priapism)	Concurrent use of lopinavir/ritonavir and sildenafil is contraindicated	Stop sildenafil if possible. If not possible, sildenafil use is an exclusion criterion for this study	Do not use sildenafil
Simvastatin	Increased risk of myopathy or rhabdomyolysis	Concomitant use of lopinavir/ritonavir with simvastatin is contraindicated	Stop simvastatin if possible. If needed, consider fluvastatin, pitavastatin or pravastatin as alternatives, because these drugs have the least potential for interaction	Do not use simvastatin. If needed, consider fluvastatin, pitavastatin or pravastatin as alternatives, because these drugs have the least potential for interaction
Atorvastatin	Atorvastatin AUC increased by 488%. Increased risk of myopathy or rhabdomyolysis	Monitor for signs of atorvastatin toxicity (rhabdomyolysis and myopathy)	Consider discontinuation of atorvastatin. If discontinuation is not possible, use with caution at the lower end of the dosing range (10–40 mg per day)	Consider alternative agents (pravastatin, fluvastatin or rosuvastatin), because these drugs have the least potential for interaction
Voriconazole	Decreased plasma concentrations of voriconazole and decreased voriconazole efficacy	Concomitant administration is contraindicated	Use alternatives to voriconazole. If no alternative exists, voriconazole use is an exclusion criterion for this study	Use alternatives to voriconazole or use with therapeutic drug monitoring. Voriconazole dose may need to be increased. If no alternative is available, discontinue lopinavir/ritonavir and continue the use of IFN-β1b. Consider another antifungal for aspergillosis
Phenytoin	Both phenytoin and ritonavir plasma concentrations may be decreased	Use with caution	Use with caution	Monitor phenytoin levels during co-administration. Adjustment of the phenytoin or fosphenytoin dose may be warranted

The information in this table was obtained from Lexicomp (http://www.wolterskluwercdi.com/lexicomp-online/) and Micromedex (https://www.micromedexsolutions.com/home/dispatch). Abbreviations: *AUC* area under the (receiver operating characteristic) curve, *CYP3A4* cytochrome P450-3A4
5. Pregnancy – eligible and consenting female participants of childbearing age will be tested for pregnancy before enrollment in the study
6. Known HIV infection, because of concerns about the development of resistance to lopinavir/ritonavir if used without combination with other anti-HIV drugs, or
7. Patient likely to be transferred to a non-participating hospital within 72 h.

### Trial intervention

Informed consent will be obtained from eligible patients or their substitute decision-makers (for patients lacking decision-making capacity). All patients who provide informed consent will be randomly allocated into one of two study groups.

#### Intervention group

The intervention group will receive the standard of care as well as lopinavir/ritonavir and recombinant IFN-β1b. Lopinavir/ritonavir (400 lopinavir mg/100 mg ritonavir) will be administered every 12 h for 14 days in tablet form. For patients who are unable to take medications by mouth, the lopinavir/ritonavir (400 lopinavir mg/100 mg ritonavir) will be administered as a 5-ml suspension every 12 h for 14 days via a pre-existing or newly placed nasogastric tube. IFN-β1b will be administered as 0.25-mg/ml subcutaneous injections on alternate days for 14 days (for a total of seven doses).

#### Control group

The control group will receive standard of care as well as placebo treatment for 14 days at the same frequency as the intervention group, to maintain blinding. One placebo will be given every 12 h and will comprise a sucrose tablet or capsule, or 5 ml of normal saline via nasogastric tube for patients who are unable to take medications by mouth. Patients in the control group will receive 1 ml of normal saline by subcutaneous injection on alternate days.

### Co-interventions

Patients in both study arms will receive standard supportive care (e.g., oral/and or intravenously administered fluids according to basal daily requirements or blood pressure support, oral or parenteral nutrition, venous thromboembolism prophylaxis, antibiotic therapy for infections, supplemental oxygen or mechanical ventilation for hypoxia or ventilation needs) at the discretion of the treating teams. Recommendations regarding current standards of care that are consistent with best clinical practices and World Health Organization guidelines will be provided to the treating teams. Data on the use of antimicrobials and corticosteroids, and aspects of respiratory, cardiovascular and renal support will be collected but not protocolized. All patients will be tested for HIV on enrollment; however, results of testing may be delayed > 24 h for some patients; therefore, we will not delay randomization while waiting HIV status. In patients who are discovered to be HIV positive, the intervention will be discontinued and outcome data will be collected as per protocol, as these patients will be included in the intention-to-treat analysis. We will have a separate on-treatment analysis that will not include these patients. Treatment of HIV infection will be as per the treating team.

### Randomization, allocation and blinding

#### Randomization

Patients will be randomly assigned to one of the two intervention arms by a variable-size block, computer-generated randomization procedure, which will ensure that the two arms contain equal numbers of patients. Randomization will be stratified according to center and according to whether the patients require mechanical ventilation (invasive or non-invasive) at the time of enrollment.

#### Allocation

Patient allocation will follow a concealed process using sealed, fully opaque, numbered envelopes.

#### Blinding

The intervention will be blinded for the research team (principal investigator, co-investigators, research coordinators and the Steering Committee), treating team (physicians, bedside nurses and clinical pharmacists), outcome assessors (including the laboratory personnel) and study patients. Pharmacists who prepare the study medication, dosing nurses (who administer the medication) and the study statistician will not be blinded. This approach was chosen because placebos that exactly match the medications in appearance are not available.

### Study drug preparation and administration

#### Storage and handling

Lopinavir/ritonavir and recombinant IFN-β1b will be stored in a secure area in the pharmacy at room temprature or refrigerator at 2–8 °C according to standard hospital procedures and the product monograph. A temperature log with daily minimum and maximum temperature readings will be kept by the site pharmacist. Any temperature excursion will be reported immediately to an unblinded project officer from the sponsor, King Abdullah International Medical Research Center, Riyadh, Saudi Arabia. The sodium chloride (0.9%) ampoules and the starch capsules will be stored in a restricted area at room temperature.

#### Study drug composition and dispensing

At patient randomization by the site investigator or by appropriately delegated study personnel, a sealed envelope containing the patient allocation will be delivered to the pharmacy. The unblinded pharmacist will open the envelope and dispense the study drug in a tamperproof sealed box to blind the treatment assignment. The sealed, opaque box will be labeled to include the following information: name and number of clinical trial protocol, patient study number and site number. The box will only be opened by the unblinded study nurse.

#### Administration

Unblinded dosing nurses, who will not be responsible for the direct clinical care of the patients, nor other study procedures including assessment of the study outcomes, will administer the treatment. A second un-blinded nurse will check the treatment regimen for each patient according to local hospital regulations for medication administration. The dosing nurses will maintain blinding by the following measures: (1) pulling the screens around the patients’ bed areas while the dose is administered and ensuring that no members of the treating team are present; (2) discarding any labels and the used syringe in a way that ensures that the identity of the medication is not revealed; and (3) avoiding any discussion regarding the study drug with other hospital staff, except the unblinded pharmacist.

#### Duration of the intervention

The study interventions will continue for 14 days according to the protocol. Medications will be discontinued in patients who are well enough to be discharged from the hospital, even if this occurs before 14 days have elapsed.

### Frequency and duration of follow-up

**Fig. 1:**
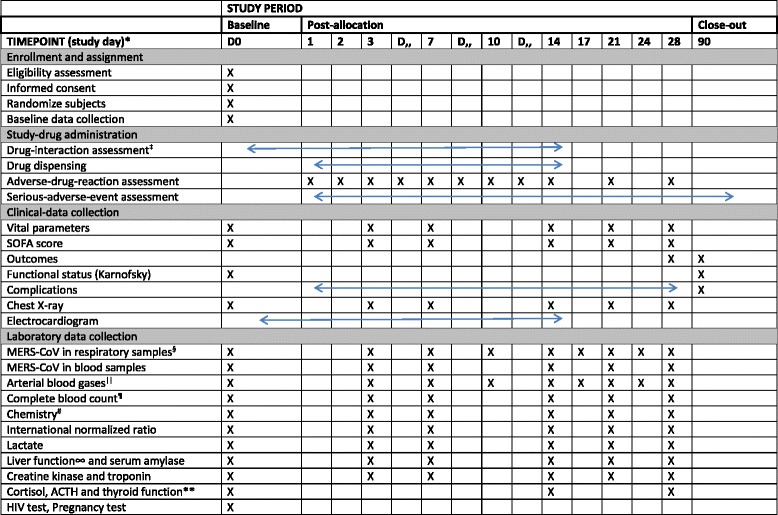
Schedule of enrollment, intervention and assessment for the MIRACLE trial according to the Standard Protocol Items: Recommendations for Interventional Trials (SPIRIT) template [27]. Arrows indicate periods of continuous or daily collection or assessment, whereas “X” indicates collection on specific days. * ± 2 days except D0 and D3. ^‡^Assessed by the clinical pharmacist. ^§^Tests on sputum, tracheal aspirate, broncheoalveolar lavage and nasopharyngeal swab to be continued twice weekly until two consecutive tests are negative. ^||^Mandatory for intubated patients, optional for non-intubated patients. ^¶^Hemoglobin, white blood cells, neutrophils, lymphocytes and platelets. ^#^Blood urea nitrogen, creatinine, potassium, sodium, chloride, bicarbonate, glucose and albumin. ∞Serum bilirubin, gamma-glutamyl transferase, alanine transaminase and aspartate aminotransferase. **Serum thyroxine, triiodothyronine and thyroid-stimulating hormone. Abbreviations: *ACTH* adrenocorticotropic hormone, *MERS-CoV* Middle East Respiratory Syndrome coronavirus, *SOFA* Sequential Organ Failure Assessment

Patients will be followed daily until discharge from hospital or day 28, whichever comes first (Fig. 1). After day 28, the patients will be followed only to document their ICU, hospital, 90-day mortality and length of stay. Collection of this information may require telephone interviews with patients or next of kin. Figure 1 shows the schedule of enrollment, intervention and assessment for the MIRACLE trial according to the SPIRIT template. The SPIRIT Checklist is given in Additional file 1.

### MERS-CoV RT-PCR testing

Diagnostic RT-PCR will be preformed per each hospital protocol in its own hospital laboratory or in the reference laboratory of the Saudi Ministry of Health. Additionally, respiratory and blood samples will be sent to King Abdullah International Research Center for semi-quantitative viral-load testing. The testing procedure includes extracting ribonucleic acid (RNA) from respiratory and serum specimens using the MagNA Pure 96 Viral NA Kit (Roche Applied Science, Indianapolis, IN, USA). The extracted nucleic acids will be tested by rRT-PCR targeting the upstream MERS-CoV envelope protein gene (upE) and open-reading frame 1a (ORF1a) regions of the MERS-CoV genome on a LightCycler 480 System (Roche Diagnostics, Mannheim, Germany) [30]. A positive control for ORF1a and upE rRT-PCR is performed according to the manufacturer’s instructions. We will document cycle thresholds (C_t_) for upE and ORF1a which indicate the start of exponential amplification. For diagnostic purposes, we consider a cycle threshold (C_t_) of < 35 the cutoff for upE and ORF1a in line with the cutoff used by the Saudi Arabia Ministry of Health reference laboratory. If C_t_ values are ≥ 35, the test will be repeated using different samples, preferably from the lower respiratory tract, to avoid false-positive results. We will use C_t_ values as semi-quantitative measures for viral RNA load.

### Study outcomes

#### Primary outcome

1. 90-day mortality

#### Secondary clinical outcomes

1. Mortality in the ICU, mortality in the hospital and 28-day mortality
2. Sequential Organ Failure Assessment (SOFA) scores at baseline and on study days 1, 3, 7, 14, 21 and 28
3. Organ support, according to the number of days within the first 28 days after enrollment when patients do not receive specific forms of support:
  - Supplemental oxygen-free days
  - Renal replacement therapy-free days
  - Vasopressor-free days
  - Invasive or non-invasive mechanical ventilation-free days
  - Organ support-free days (that is, days free of invasive mechanical ventilation, renal replacement therapy and vasopressors)
  - Extracorporeal circulation support-free days
4. ICU-free days and hospital length of stay:
  - ICU-free days are the number of days when patients are not being cared for in the ICU during the first 28 days after enrollment. Patients who die within 28 days will be assigned the value “0”
  - The hospital length of stay will be calculated as the number of days between admission and discharge from the hospital. Because of the competing risk effect of death on length of stay, length of stay will be also reported for survivors alone

#### Secondary laboratory outcomes

1. Viral replication kinetics will be calculated by determining viral RNA loads as reflected by C_t_ values by semi-quantitative PCR in serial samples of upper and lower respiratory tract secretions and blood, collected twice per week. Quantitative PCR, which may be more sensitive than C_t_ values, will be used if it becomes available during the trial
2. Time to clearance from the lower respiratory tract will be determined from the results of RT-PCR analyses of lower respiratory tract secretions or nasopharyngeal swabs in non-intubated patients who are unable to give sputum samples; clearance will be defined as two negative RT-PCR results not followed by a positive one. Patients who die before clearance will be censored at the time of death
3. Planned sub-studies will examine cytokine, chemokine and immune responses in serially collected blood samples. A detailed protocol of this sub-study will be published separately

#### Safety outcomes

1. Safety outcomes will be assessed from serious adverse event reports. The following serious adverse events will be documented at any time during the study period:
  - acute pancreatitis (defined as having two of the following three features: (1) abdominal pain consistent with acute pancreatitis; (2) serum lipase or amylase at least three times greater than the upper limit of normal; and (3) characteristic findings of acute pancreatitis on contrast-enhanced computed tomography) [31] or magnetic resonance imaging
  - elevation of ALT to more than five-fold upper normal limit
  - anaphylaxis
  - bleeding diathesis (INR > 3 without anticoagulant therapy)
2. The following data regarding adverse drug reactions will be assessed with the treating team and recorded daily for 14 days and then at 21 days and 28 days after enrollment:
  - Allergic or sensitivity/hypersensitivity reactions, including rash, urticaria, tongue edema, bronchospasm, dyspnea and skin necrosis at the injection site
  - Gastrointestinal signs or symptoms, including nausea, vomiting, abdominal pain and diarrhea
  - Neurological symptoms, including fatigue, headache, insomnia, psychosis, depression and mania

#### Functional outcomes

Data will be collected at baseline before the current illness and at 90 days and may require telephone interviews with the patients or next of kin to determine the patients’ status according to the Karnofsky Performance Scale, which is a scale from 100 (indicating “Normal,” no complaints; no evidence of disease) to 0 (indicating death) [32].

### Safety measures

#### Drug interactions

A clinical pharmacist and the treating physician will evaluate each patient at the time of enrollment and daily throughout the study period until day 14 to identify any interactions between the investigated treatments and other drugs, and they will take action accordingly. Table 1 summarizes common medications that may interact with lopinavir/ritonavir and a suggested action plan.

#### Liver function tests

Elevated liver enzymes are common in MERS infection due to the liver involvement in the disease. Based on analysis of our historical data, liver enzymes were not more elevated in patients treated with ribavirin and IFN-α. Based on HIV literature, aminotransferase elevation levels (more than five times the upper limit of normal) occur in 3 to 10% of patients taking lopinavir/ritonavir-containing antiretroviral regimens [33]. These laboratory abnormalities are usually asymptomatic and self-limited and often resolve even without discontinuation of the drug [33]. Clinically apparent hepatotoxicity due to lopinavir/ritonavir occurs, but is rare, with symptoms or jaundice appear usually after 1 to 8 weeks of starting therapy [33]. The hepatotoxicity is usually self-limited; although fatal cases have been reported [33]. The treating team should discontinue or minimize concomitant use of any medication that has hepatotoxic potential.

#### Rules regarding premature discontinuation of treatment

The study intervention will be suspended if the subject develops any of above defined serious adverse events occurred: (1) acute pancreatitis; (2) elevation of ALT to more than five-fold the upper normal limit; (3) anaphylaxis; and (4) bleeding diathesis. These events will be reviewed by at least two people with a third as necessary, all of whom are blinded to the intervention allocation, to adjudicate these events by looking at the study data/clinical records. In case of premature discontinuation of treatment, other aspects of the study procedures, such as data collection and laboratory testing, will be continued and patient data will be included in the intention-to-treat analysis.

#### Unblinding

Breaking of blinding can be occur in the event that the treating team believes that there is a very strong reason to know the study drug allocation and after discussion with the study principal investigator. In all such cases, the adverse event necessitating emergency unblinding will be reported as a serious adverse event.

#### Serious adverse events

Any unexpected, medical occurrence that results in death, prolonged hospitalization, persistent or significant disability or incapacity, which is judged to be causally related to the study intervention, will be reported as a serious adverse event to the Institutional Review Board and the Data Safety Monitoring Board. Potential serious adverse events for the trial are defined above and include (1) acute pancreatitis; (2) severe elevation of ALT to more than five-fold the upper normal limit; (3) anaphylaxis; and (4) bleeding diathesis.

### Study management

#### Steering Committee

The Steering Committee, led by the principal investigator, will be responsible for overseeing the conduction of the trial, providing training for new sites, ensuring compliance with the study procedures, addressing challenges that occur at all sites, reviewing serious adverse events and formulating the statistical analysis plan.

#### Data Safety Monitoring Board

The Data Safety Monitoring Board, which is responsible for reviewing reports regarding the safety of the study patients and protocol adherence, may make recommendations to continue or terminate the study on the basis of the results from the interim analysis. The board will meet at the beginning of the study and at 6-monthly intervals thereafter, or as needed.

#### Quality control and assurance

Several procedures to ensure data quality and protocol compliance are undertaken including: (1) Training sessions for the study team in the participating centers prior to study commencement; (2) Startup meetings for all sites, either on site or remotely; and (3) Monitoring visits before, during and at the close out of the study.

#### Study drug distribution to other centers

The Investigational Drug Unit at King Abdullah International Medical Research Center will distribute the study medication and the placebo to other centers. Randomization and blinding will be performed at the local pharmacy of each hospital according to the procedures described above.

### Ethical issues

The MIRACLE study is approved by the Scientific Committee and the Institutional Review Boards of the participating sites and registered at the Saudi Food and Drug Authority (SFDA).

The study will be conducted in accordance with the ethical principles of the Declaration of Helsinki and the International Council for Harmonization–Good Clinical Practice guidelines. Informed consent will be obtained from patients or their substitute decision-makers. Site invistigators will explain the objectives of the trial and its potential risks and benefits to patients when possible, or to substitute decision-makers, during the process of obtaining consent. No compensation is provided for enrollment in the trial.

Patient personal data are de-identified. Data from all centers will be stored on a secure place at King Abdullah International Medical Research Center, Riyadh. A list of names/hospital numbers and unique study identification (ID) will be kept in a separate location to the trial data, which has only the associated unique subject ID attached to it.

### Statistical analysis

Baseline clinical and demographic characteristics will be summarized and reported using descriptive statistics. Categorical variables will be reported as numbers and percentages, and will be compared using the chi-square test or Fisher’s exact test, as appropriate. Continuous variables will be reported as means and standard deviations or as medians and interquartile ranges, and will be compared using Student’s *t* test or the Mann-Whitney *U* test, as appropriate.

The primary analysis will be conducted according to the intention-to-treat principle and will be unadjusted. Per-protocol analysis will be performed and reported separately. The primary analysis will examine the difference in primary outcome between groups and will be reported as absolute risk reduction, with a 95% confidence interval. We do not anticipate imbalances between the two groups. Secondary analysis will be conducted using multiple logistic regression analysis in which death within 90 days will be modeled as the dependent variable and a set of baseline variables that are strongly believed to affect the outcome of MERS-CoV infection will be included as independent variables. Those variables will include at minimum age, community-acquired versus hospital-acquired infection, mechanical ventilation, center, and SOFA score [34]. The multivariable analysis will also include clinically important and unequally distributed baseline variables between the two groups (*p* < 0.05). Median survival time will be summarized and reported using Kaplan-Meier curves and will be compared between the study groups using the log-rank test. Statistical tests for variables other than the primary outcome will be performed using a two-sided alpha value of 5% to denote significance level.

If patient numbers permits (e.g., no fewer than five patients in subgroups of interest), stratified analysis will be carried out between the following subgroups:

1. Patients with and without mechanical ventilation at the time of randomization
2. Patients with different Acute Physiology and Chronic Health Evaluation II (APACHE II) disease-severity scores at the time of randomization stratified to high (above median) and low (below median) APACHE II scores
3. Patients receiving and those not receiving vasopressors at the time of randomization
4. Patients with ≤ 7 and > 7 days between onset of symptoms to enrollment
5. Patients receiving and those not receiving renal replacement therapy at randomization

### Interim analysis and continuous study planning

**Fig. 2:**
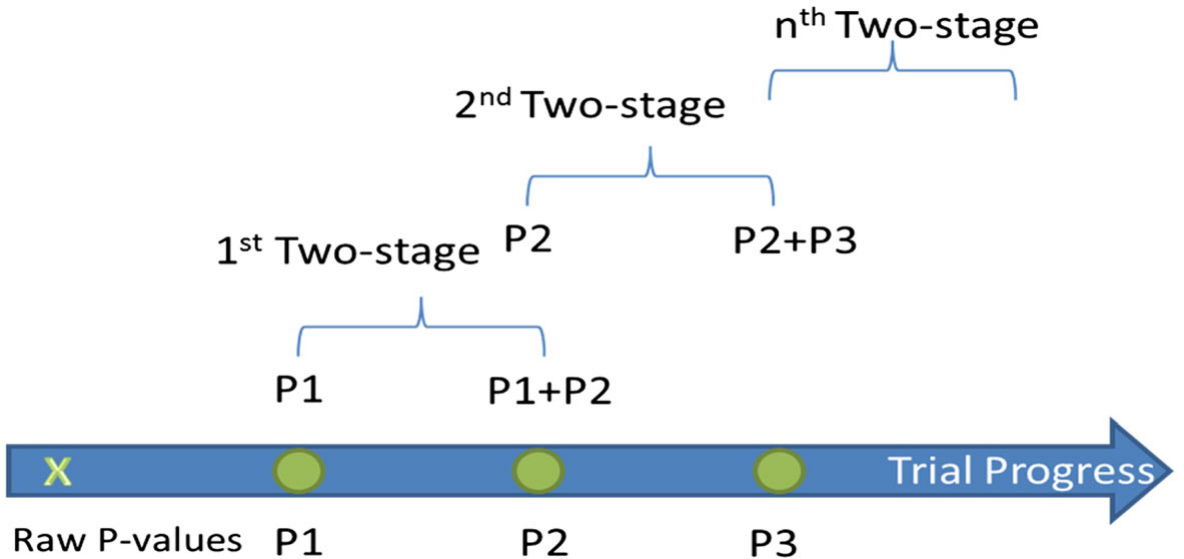
Schematic illustration of the MIRACLE trial design with 2 two-stage components with two interim analyses and one final analysis. Raw *p* values are obtained from the one-sided chi-square for difference in proportion at each interim analysis

Because of the uncertainty surrounding the recruitment rate and the efficacy level of the treatment, the trial is designed as recursive, two-stage, group sequential randomized trial [28]. The trial is designed initially to have 2 two-stage components with two interim analyses and one final analysis (Fig. 2). The first two-stage component is designed to determine futility stopping and adjust sample size, but not efficacy stopping. The second two-stage component is designed to determine efficacy stopping and possibly readjustment of sample size to maintain conditional power at the final analysis. A classic two-group design requires a total of 194 subjects (97 subjects per group) to have a 80% power at a significant level of 2.5% (one-sided test) to detect 20% absolute risk reduction in 90-day mortality among subjects receiving treatment (20%) compared to the control group (40%). The trial will start the first two-stage design with 136 subjects (68 subjects per group). We will conduct the first interim analysis when the total subject with 90 days of follow-up reaches 34 subjects (17 per group), which is about 17.5% of the total sample size needed for classical design. We use the method of summing of stage-wise *p* value to determine the one-sided stopping boundaries in the first two-stage design listed in Table 2. At the first interim analysis we will use the stage-wise *p* values obtained from the chi-square test for difference in proportion to determine whether the trial will be stopped for futility or not. Should the trial continue, sample size re-estimation based on the observed effect size will be determined using the following formula assuming a conditional power of 80%:

**Table 2:** Stopping boundaries

Boundary	Value	Comment
Efficacy stopping boundary (α_11_)	0	No stopping for efficacy
Efficacy stopping boundary (β_11_)	0.2	Stop the trial for futility if less than stage-wise *p* value
Efficacy stopping boundary (α_12_)	0.2250	Stop trial for efficacy at the second stage or recalculate based on conditional power at first interim analysis

$$ n12={\left[\frac{\sqrt{2}\sigma }{\delta}\left({\theta}^{-1}\left(1-\alpha 12+p11\right)-{\theta}^{-1}\left(1- Pc\right)\right)\right]}^2 $$

Based on the estimated sample size, we will recalculate the conditional error and set the parameters for the second of the two stages. At the second interim analysis, should the trial continue for efficacy, sample size readjustment will be made based on the previous formula and new boundaries will be calculated for the final-stage analysis.

Upon completion, the results of the trial is planned to be submitted to peer-reviewed journal. Authorship will follow the Uniform Requirements for Manuscripts Submitted to Biomedical Journals [35].

## Discussion

This study will be the first randomized controlled trial of a treatment for MERS-CoV infection. Although this type of study provides the best method for determining treatment efficacy during disease outbreaks [36, 37], conducting a randomized controlled trial is logistically challenging [37, 38] since the distribution of MERS cases is sporadic and unpredictable, both geographically and temporally, necessitating the involvement of several centers in the trial to ensure adequate recruitment. This requirement contributes greatly to the planning and administration associated with the trial.

To overcome the uncertainty in recruitment rate and efficacy of the trial’s treatment we designed the trial using recursive, two-stage adaptive design, which is a relatively new method for group sequential trials [28]. The approach is based on the conditional error principle which allows for continuous adjustment of the trial parameters using data observed during prior stages without inflating type I error [39]. Another advantage of this method is the flexibility in setting the timing and the number of needed interim analyses. Such flexibility is necessary in situations where recruitment rate is unpredictable and sudden flux in patient recruitment could happen at any time.

For many emerging infectious diseases, including MERS, patients are often treated with therapeutics on the basis of minimal evidence. For example, patients with MERS have been treated with ribavirin and IFN-α despite the lack of clinical trials exploring the effectiveness of this treatment combination. If these patients had been included in a properly designed study, conclusive evidence might have been generated. Performing such a trial meets a critical need.

Some limitations to our study design should be noted. Complete blinding was not feasible because of the unavailability of appropriate placebo; therefore, the study is unblinding to the dosing nurses and the dispensing pharmacist, both of whom are not otherwise involved in the patient care. However, blinding will be maintained to all other members of the clinical and research team. Because of the sporadic and unpredictable nature of the disease, the number of patients from some sites may be low.

### Trial status

The MIRACLE trial has already been approved by the Institutional Review Boards at eight hospitals and by the Scientific Advisory Board at the Ministry of Health in Saudi Arabia and the Saudi Food and Drug Authority. Enrollment for this study began in November 2016, and has enrolled thirteen patients as of Jan 24-2018. At present, eight sites have completed the regulatory requirements and are actively screening for eligible patients; 12 other centers are in progress.

## Additional file

Additional file 1: SPIRIT 2013 Checklist: recommended items to address in a clinical trial protocol and related documents*. (DOC 120 kb)

## Abbreviations

ALT: Alanine aminotransferase
APACHE II: Acute Physiology and Chronic Health Evaluation II
Ct: Threshold cycle
EC: Effective concentration
HIV: Human immunodeficiency virus
IFN: Interferon
IFN-α: Interferon alpha
IFN-β1b: Interferon beta-1b
MERS-CoV: Middle East Respiratory Syndrome coronavirus
MIRACLE trial: the *M*ERS-CoV *I*nfection t*R*eated with *A C*ombination of *L*opinavir/ritonavir and int*E*rferon β1b
MMF: Mycophenolate mofetil
RT-PCR: Reverse-transcription polymerase chain reaction
SARS: Severe Acute Respiratory Syndrome
SPIRIT: Standard Protocol Items: Recommendations for Interventional Trials
